# From Daily Routines to Cardiovascular Health: The Untapped Potential of Incidental Physical Activity

**DOI:** 10.31083/RCM46408

**Published:** 2026-01-09

**Authors:** Yangyang Huang, Lingyun Luo, Xuelian Luo, Le Zhang, Cuntai Zhang

**Affiliations:** ^1^Department of Geriatrics, Tongji Hospital, Tongji Medical College, Huazhong University of Science and Technology, 430030 Wuhan, Hubei, China; ^2^Key Laboratory of Vascular Aging, Ministry of Education, Tongji Hospital, Tongji Medical College, Huazhong University of Science and Technology, 430030 Wuhan, Hubei, China; ^3^Department of Cardiology, Tongji Hospital, Tongji Medical College, Huazhong University of Science and Technology, 430030 Wuhan, Hubei, China; ^4^Hubei Provincial Engineering Research Center of Vascular Interventional Therapy, 430030 Wuhan, Hubei, China; ^5^Department of Oncology, The Third Affiliated Hospital of Chongqing Medical University, 401120 Chongqing, China

**Keywords:** motor activity, life style, cardiovascular diseases, primary prevention, exercise

## Abstract

Cardiovascular diseases (CVDs) represent the primary cause of death worldwide, thereby demanding effective preventive measures. Incidental physical activity (IPA), which encompasses non-exercise movements naturally integrated into daily routines, offers a practical and promising strategy for reducing CVD risk. Research, particularly from the UK Biobank, has consistently highlighted the cardiovascular advantages of IPA across diverse populations. However, systematic guidelines for integrating IPA into cardiovascular care are limited. Thus, this review aims to provide a comprehensive synthesis of IPA, including a definition, classification by intensity, current evidence linking IPA to cardiovascular health, and the underlying mechanisms involved. Present research underscores the extensive benefits of IPA, particularly the pronounced effectiveness of vigorous IPA (VIPA). However, approaching these findings with caution is essential, especially considering the limited representation of individuals predisposed to exercise-induced sudden cardiac events in middle-aged and older cohorts. Therefore, while the advantages of IPA are clear, further investigation is warranted to understand the implications of IPA for all populations. In conclusion, we recommend integrating IPA as a complementary strategy alongside structured exercise in clinical practice. However, emphasizing risk mitigation strategies for VIPA is crucial, particularly for at-risk individuals. This review aims to provide practical guidance on the effective implementation of IPA in cardiovascular health management.

## 1. Introduction

Cardiovascular diseases (CVDs), such as ischemic heart disease and stroke, remain the foremost cause of death globally, responsible for approximately one-third of all deaths worldwide [1, 2, 3]. An estimated 330 million individuals in China are currently affected by CVDs [4]. Accelerated population aging, demographic growth, and the prevalence of sedentary lifestyles have contributed to a consistent rise in the incidence and mortality rates of CVD worldwide over the past thirty years [5, 6]. Annual CVD-related mortality is projected to rise to 35.6 million by 2050, compared to 20.5 million in 2025 [7].

Conventional exercise programs frequently struggle with long-term adherence, highlighting the necessity for additional preventive strategies. In response, recent research has explored the impact of incidental physical activity (IPA)—which refers to spontaneous, non-exercise physical activities that are seamlessly woven into daily life without specific fitness objectives, such as household chores, active commuting, and movement related to work—on enhancing cardiovascular health [8, 9, 10, 11, 12]. IPA encompasses a broad spectrum of intensities, ranging from light to vigorous, and can occur in short bursts or extend over longer durations. Epidemiological studies indicate a negative correlation between IPA and all-cause mortality, highlighting its effectiveness as a practical and accessible approach to preventing cardiovascular diseases by encouraging regular energy expenditure [10, 12, 13]. Proposed mechanisms through which IPA may contribute to cardiovascular health include the enhancement of cardiorespiratory fitness, improved metabolic regulation—such as better glycemic control and lipid homeostasis—and the reduction of systemic inflammation.

Despite the increasing evidence supporting the cardiovascular benefits of IPA, there is a notable lack of clear and systematic guidelines for its broader integration into clinical practice and everyday routines. This review seeks to promote the widespread adoption of IPA as a preventive strategy for cardiovascular health. It explores five essential aspects: (1) the definition of IPA, (2) its classification according to intensity, (3) its cardiovascular benefits, (4) the potential mechanisms that contribute to these benefits, and (5) strategies to enhance public awareness and encourage the deliberate inclusion of IPA in daily life, considering both clinical and public health applications.

## 2. Topics and Results

### 2.1 Conceptual and Operational Considerations of IPA

Despite broad recognition of IPA as an important aspect of physical activity, there remains a need for greater clarity concerning its behavioral scope and the influence of intentionality on its classification. Traditionally, many studies have characterized IPA as unstructured, unintentional, and not specifically aimed at exercise or health improvement [10]. In recent years, however, growing evidence on the health benefits of IPA has influenced physical activity guidelines. For example, the World Health Organization moved away from recommending “at least 10 continuous minutes” of activity toward acknowledging that “any amount of physical activity is better than none, and the more, the better” [14]. This transition reflects an important evolution: IPA is now promoted intentionally for health, even though it was originally conceived as a non-purposeful activity. Terminological variations persist in the literature, where phrases such as “incidental physical activity”, “non-exercise activity thermogenesis”, and “vigorous incidental physical activity” are often used alongside “activities of daily living” [10]. While these terms emphasize different aspects, the fundamental idea of IPA—non-structured movement integrated into everyday life—remains consistently acknowledged. Consequently, the challenge is not in defining IPA itself but in operationalizing its various subtypes, particularly distinguishing between unintentional and intentionally integrated IPA.

To effectively capture intentionality in practice, various approaches can be utilized in both research and public health settings. In epidemiological surveys, questionnaires can assess intent by asking questions such as, “Do you typically take the stairs to increase your physical activity?” or “How often do you opt for active transportation (e.g., walking or cycling) for health reasons?” In device-based assessments, such as those using accelerometers, the volume and intensity of activity can be objectively measured during brief intervals, with intentionality further inferred through supplementary tools like ecological momentary assessment. For example, smartphone prompts could inquire, “Was this activity performed for exercise or health purposes?” However, in public health messaging, the distinction between intentional and unintentional IPA is often less critical, as the primary focus is on encouraging behavioral integration irrespective of the initial motivation. Messages like “Choose stairs whenever possible” promote intentional action without emphasizing underlying motivation.

We suggest defining IPA based not on intentionality but on its behavioral context: as unstructured movements inherent to daily routines, including household chores, active commuting, and occupational tasks. This perspective eliminates ambiguity and aligns with modern health strategies. For instance, whether stair climbing occurs due to a broken elevator (unintentional) or a health-driven choice (intentional), it is still classified as IPA. This cohesive definition enhances communication and encourages individuals to consciously incorporate more IPA into their daily lives, transforming incidental movements into their daily lives, transforming opportunities for improving health.

### 2.2 Physiological Classification of IPA

IPA is operationally distinguished from structured exercise by its integration into daily routines through short, intermittent bouts interspersed with rest or low-intensity movement [15]. In alignment with the Compendium of Physical Activities [16], IPA has been categorized into various domains, including household tasks, gardening, ambulation, and cycling [17].

The existing literature presents various classifications and stratifications of IPA, with specific criteria outlined in Table 1 (Ref. [8, 9, 10, 11, 12]).

**Table 1.** **Heterogeneity in classification criteria for IPA across recent literature**.

Literature	Classification criteria
Cao *et al*., 2025 [8]	accelerometer-derived intensity
Reyes-Molina *et al*., 2025 [10]	systematic analysis of 55 articles using the FITT principle (Frequency, Intensity, Time, and Type—though Frequency was deemed less relevant for IPA)
Koemel *et al*., 2025 [9]	standard metabolic equivalent of task (MET) threshold derived from accelerometer data, with/without bout duration restriction
Stamatakis *et al*., 2025 [11]	standard MET derived from accelerometer data without explicit mention of bout duration
Lee and Jung, 2025 [12]	intensity based on wrist-worn accelerometer-derived MET thresholds and specific activities/postures

Abbreviations: MET, metabolic equivalent of task; IPA, incidental physical activity.

**Fig. 1.**
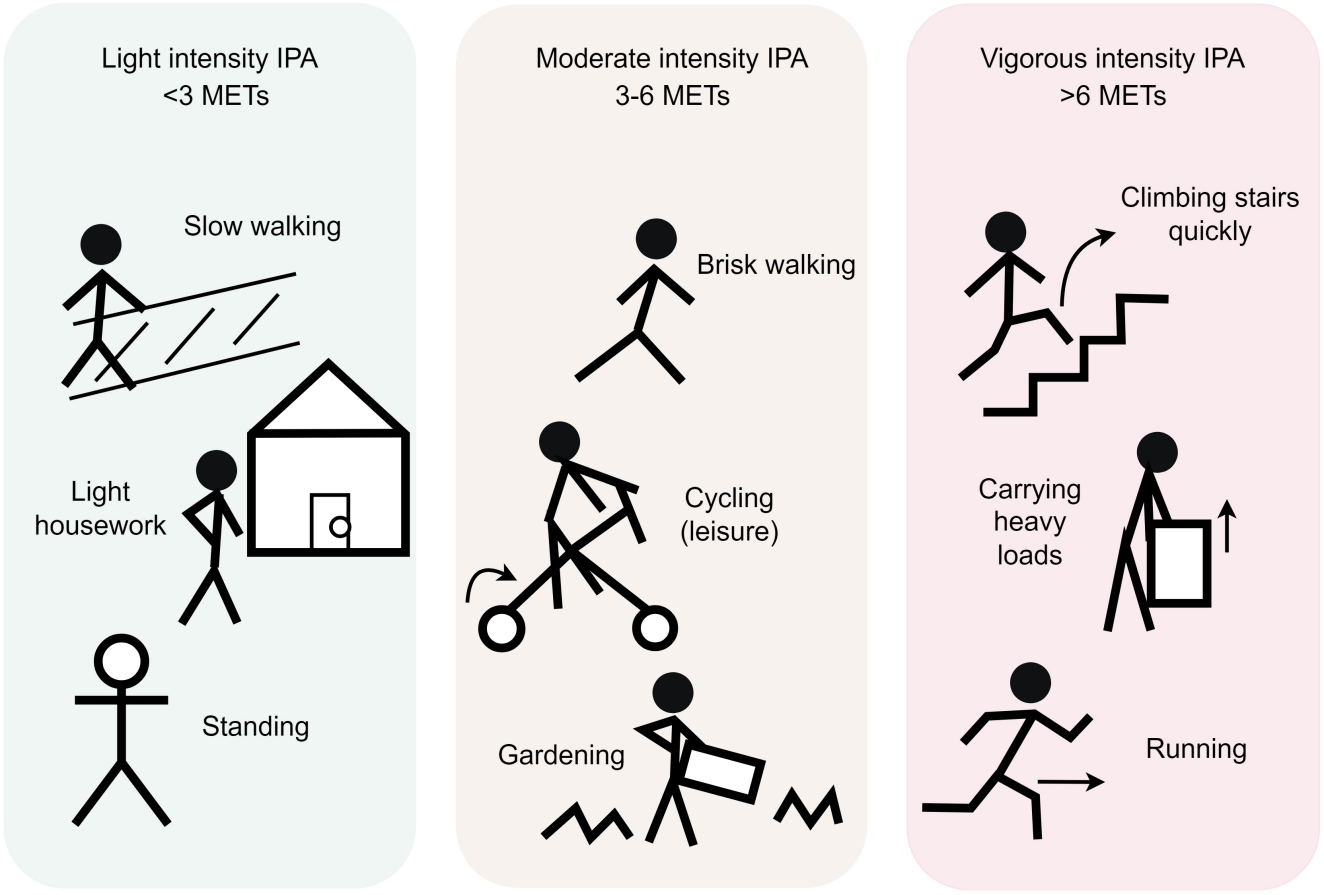
**Classification of IPA**. IPA can be classified into three categories. Light intensity IPA (LIPA): characterized by MET values <3.0 or step cadence ≤19 steps/minute [9], such as leisurely walking, light stretching, or light standing tasks (e.g., washing dishes, folding laundry). Moderate intensity IPA (MIPA): defined by METs 3.0–5.9 or cadence 20–39 steps/minute [9], including brisk walking for transportation, gardening, carrying light loads, cycling, or climbing stairs. Vigorous intensity IPA (VIPA): Vigorous-intensity activities (≥6 METs) performed in ultrashort bursts (≤1–3 minutes), exemplified by vigorous intermittent lifestyle physical activity (≤1-minute bouts, e.g., sprinting upstairs) or moderate-to-vigorous intermittent lifestyle physical activity (MV-ILPA, ≤3-minute bouts, e.g., carrying groceries). The diagram was drawn by Figdraw. Abbreviations: IPA, incidental physical activity; LIPA, light intensity IPA; MET, metabolic equivalent of task; MIPA, Moderate intensity IPA; MV-ILPA, moderate-to-vigorous intermittent lifestyle physical activity; VIPA, vigorous intensity IPA.

These classifications are typically based on objective metrics such as accelerometer-derived milligravity thresholds [8], step cadence [10], bout duration, metabolic equivalent of task (MET) thresholds, cardiorespiratory demands, and neuromuscular engagement [8]. Activities are categorized into several types: sedentary behavior, standing utilitarian tasks, walking, and high-energy activities like running. To pinpoint specific activities and their corresponding intensity levels, researchers frequently use a validated two-stage random forest classifier, which boasts an overall accuracy of 86.4% [11, 18]. Based on these criteria, IPA can be stratified into three distinct categories, as shown in Fig. 1 (Ref. [9]) and Table 2.

**Table 2.** **Classification of IPA by intensity**.

IPA classification	MET values	Step cadence	Energy expenditure	Heart rate and VO_2_	Examples
LIPA	<3.0 METs	≤19 steps/minute	30–125 milligravities	minimal increases	leisurely walking, standing conversations, light housework
MIPA	3.0–5.9 METs	20–39 steps/minute	125–400 milligravities	40–60% of heart rate reserve or 64–76% of maximal heart rate	brisk walking, gardening, carrying light loads, cycling
VIPA	≥6 METs	/	>400 milligravities	VO_2_ >60% of peak levels	sprinting upstairs, carrying groceries, rapid ambulation

The MET-based intensity thresholds for IPA are consistent with standard classifications for physical activity, but are applied here to identify short, intermittent bouts of movement inherent to daily routines. Abbreviations: IPA, incidental physical activity; LIPA, light intensity IPA; MET, metabolic equivalent of task; MIPA, moderate intensity IPA; VIPA, vigorous intensity IPA; VO_2_, oxygen uptake.

Light intensity IPA (LIPA): Characterized by MET values <3.0 or step cadence ≤19 steps/minute [10], such as leisurely walking, light stretching, or light standing tasks (e.g., washing dishes, folding laundry), with energy expenditure between 30–125 milligravities [8]. LIPA induces minimal increases in heart rate and oxygen uptake (VO_2_) without significant lactate accumulation [8, 19]. It is characterized by energy expenditure below 40% of HR reserve and is frequently integrated into daily activities as unstructured movement. These movements mainly serve to break up sedentary patterns but do not provide enough intensity to induce substantial cardiometabolic adaptations [9, 20]. They are often fragmented into brief, intermittent bouts [21].

Moderate intensity IPA (MIPA): Defined by METs 3.0–5.9 or cadence 20–39 steps/minute [10], including brisk walking for transportation, gardening, carrying light loads, cycling, or climbing stairs, with energy expenditure between 125–400 milligravities [8], achieving 40–60% of heart rate reserve or 64–76% of maximal heart rate [14]. This intensity boosts aerobic capacity, enhances metabolic efficiency, and is associated with significant reductions in both systolic blood pressure and low-density lipoprotein cholesterol levels [8]. Accumulating ≥21.8 minutes/day of such activities enhances cardiorespiratory fitness and metabolic health [9, 14].

Vigorous intensity IPA (VIPA): Vigorous-intensity activities (≥6 METs) performed in ultrashort bursts (≤1–3 minutes), exemplified by vigorous intermittent lifestyle physical activity (≤1-minute bouts, e.g., sprinting upstairs) or moderate-to-vigorous intermittent lifestyle physical activity (MV-ILPA, ≤3-minute bouts, e.g., carrying groceries), with energy expenditure >400 milligravities [8]. VIPA is episodic but confers disproportionate metabolic benefits [8], and demonstrates pronounced dose-response associations with health outcomes [22], driving VO_2_ to >60% of peak levels and stimulating anaerobic glycolysis [23]. It triggers quick cardiovascular changes, such as enhanced myocardial oxygen consumption and decreased arterial stiffness. However, it necessitates vigilant monitoring because of the increased hemodynamic stress involved [8]. Even minimal durations (e.g., 4.1 minutes/day of VIPA) yield significant cardiovascular protection [21, 24].

### 2.3 Cardiovascular Benefits of IPA

Emerging epidemiological evidence suggests that IPA, even at light intensity, offers substantial cardiovascular protection across various populations. Notably, the benefits of IPA tend to increase significantly with greater intensity (Table 3, Ref. [8, 9, 11, 25]). This section systematically examines the cardiovascular protective effects of IPA through recent device-measured data and clinical studies.

**Table 3.** **Summary of research findings on the impact of IPA on cardiovascular health: data from the UK Biobank**.

Article Title	Journal	Follow-up Time	Study Population	Outcome Measures	Study Conclusions
Dose Response of Incidental Physical Activity Against Cardiovascular Events and Mortality [11]	Circulation	Mean 7.9 ± 1.0 years (ended Nov 30, 2022).	24,139 non-exercising, middle-aged and older UK adults (mean age 61.9 ± 7.6 years, 56.2% women).	MACE; CVD mortality; All-cause mortality	4.6 min/day vigorous IPA reduces CVD death by 38%.
Can incidental physical activity offset the deleterious associations of sedentary behaviour with major adverse cardiovascular events? [9]	European Journal of Preventive Cardiology	Median 8.0 years (ended Nov 30, 2022).	22,368 non-exercising middle-aged and older UK adults (median age (IQR): 62.9 (56.2, 67.7) years, 41.8% male).	MACE	4 min/day vigorous IPA offsets sedentary-related MACE risk.
Association of accelerometer-derived physical activity with all-cause and cause-specific mortality among individuals with cardiovascular diseases: a prospective cohort study [8]	European Journal of Preventive Cardiology	Median 6.8 years (ended Nov 12, 2021).	8024 adults with pre-existing CVDs (mean age 66.6 years; 34.1% female).	All-cause mortality; Cancer mortality; CVD mortality	15 min/week vigorous IPA reduces all-cause mortality by 50% in CVD patients.
Prospective Associations of Daily Step Counts and Intensity With Cancer and Cardiovascular Disease Incidence and Mortality and All-Cause Mortality [25]	JAMA Internal Medicine	Median 7 years (baseline: 2013–2015; outcome ascertainment through October 2021).	78,500 adults aged 40–79 years (mean age: 61 years, SD: 8 years; 55% female, 97% White).	All-cause mortality; Cancer mortality; CVD mortality; Cancer incidence; CVD incidence	Higher daily step counts (up to 10,000 steps) and stepping intensity (peak-30 cadence) were prospectively associated with reduced all-cause CVD and cancer mortality/incidence over 7 years.

Abbreviations: CVD, cardiovascular disease; MACE, major adverse cardiovascular events, including incident stroke, myocardial infarction, heart failure, or CVD death; IQR, Interquartile Range; SD, Standard Deviation.

Objective monitoring studies utilizing wearable devices demonstrate that even sporadic bouts of vigorous intermittent lifestyle physical activity, e.g., stair climbing, provide clinically significant health benefits. This evidence supports the adoption of “lifestyle-integrated” preventive approaches [25]. Current World Health Organization recommendations (75–150 minutes/week of moderate-to-vigorous activity) [14] can be effectively adapted to IPA frameworks.

Stamatakis *et al*.’s analysis [11] of UK Biobank data revealed that both MIPA and VIPA significantly lower the risk of major adverse cardiovascular events (MACE) and CVDs mortality in middle-aged and older adults who do not engage in regular exercise. The findings indicated that a median daily duration of 23.8 min of MIPA or 4.6 minutes of VIPA corresponded with a 40–50% reduction in MACE risk and a 50% decrease in CVD mortality [11]. The study established novel inter-intensity “health equivalence”: 1 minute of VIPA corresponded to 2.8 (for MACE) to 3.4 (for CVD mortality) minutes of MIPA for CVD outcomes, and 34.7–48.5 minutes of light IPA (LIPA) [11]. These findings challenge traditional MET-based assumptions (presumed 1:2 VIPA: MIPA ratio) and emphasize intensity-specific cardioprotective mechanisms [26]. Further investigations revealed that brief VIPA bouts (>4.1 min/day) negated sedentary-associated MACE risk (>11.4 h/day) in non-exercisers [9], while accumulating 31–65 min/day MVPA or 26–52 min/day MV-ILPA neutralized sedentary-related mortality [22, 24].

In populations with pre-existing CVDs, we observed an inverse association between the IPA volume and all-cause mortality. The mortality risk decreased by 50% with ≥1380 min/week LIPA (hazard ratio (HR): 0.63; 95% confidence interval (CI): 0.51–0.79), whereas ≥155 min/week MIPA yielded a 58% reduction (HR: 0.42) [8]. Notably, ≥45 min/week VIPA reduced CVD-specific mortality by 54% versus inactivity [8]. According to population-attributable fraction (PAF) estimates, inadequate engagement in VIPA was responsible for 43% of preventable CVD deaths, markedly exceeding the proportions associated with insufficient moderate-intensity (18.1%) and light-intensity (9.8%) activity [8]. VIPA demonstrated superior mortality risk reduction (PAFs up to 52.1%) compared to longer-duration moderate activities, likely mediated through rapid metabolic adaptations [21, 27]. Analysis of the objective measurements in an accelerometer study further revealed that the light-intensity stepping (<40 steps/min) reduced CVD mortality risk (HR 0.90 per 10% daily increase), while higher cadences (peak-30 cadence) conferred greater protection (HR 0.86) in adults aged 40–79 without baseline CVDs [25].

Besides its systemic benefits, physical activity has been shown to elicit highly individualized, intensity-dependent hemorheological adaptations [28]. These include improved red blood cell flexibility and reduced aggregation, lowered blood viscosity, and modified plasma levels of fibrinogen, albumin, and other rheologically active components [28]. Exercise also expands plasma volume and total blood volume, particularly in trained individuals [29], and induces functional and structural endothelial adaptations—though response thresholds for markers such as endothelin-1 are person-specific [30]. Additional benefits involve lowered blood pressure via upregulation of nitric oxide and prostaglandins [31, 32], and hypoxia-induced improvements in blood flow properties [33, 34]. These micro-level changes in hematology and hemorheology, influenced by the frequency, intensity, and duration of physical activity, are fundamental to the cardiovascular benefits associated with regular exercise [28].

### 2.4 Potential Mechanisms Underlying IPA Benefits

The cardiovascular advantages of IPA arise from interconnected biological mechanisms. Improved endothelial function, indicated by increased nitric oxide bioavailability at all IPA intensities, mitigates atherogenic drivers, such as oxidative stress and inflammatory responses [15, 35]. This vascular improvement is accompanied by stabilized autonomic regulation, in which augmented parasympathetic tone and heart rate variability reduce arrhythmia susceptibility [36]. At the metabolic level, IPA stimulates mitochondrial oxidative phosphorylation efficiency—particularly in fatty acid metabolism—through AMP-activated protein kinase/Peroxisome proliferator-activated receptor gamma coactivator 1-alpha (AMPK/PGC-1α) signaling cascades that upregulate carnitine palmitoyltransferase I (CPT1) expression and adenosine triphosphate (ATP) synthesis, counteracting metabolic rigidity in cardiac tissue [8, 15, 37]. Furthermore, regular daily activity, such as that achieved through IPA, is crucial for maintaining metabolic health by enhancing postprandial fat metabolism [38] and preventing the resistance to metabolic improvements that can follow periods of inactivity [39]. In addition, IPA inhibits systemic inflammation via intensity-dependent mechanisms: vigorous IPA primarily boosts endothelial nitric oxide synthase (eNOS) activation to reduce arterial stiffness, whereas moderate IPA decreases the NAIP, CIITA, HET-E, and TP1 (NACHT), Leucine-Rich Repeat (LRR), and PYD domains-containing protein 3 (NLRP3) inflammasome activity (↓Interleukin-1 beta (IL-1β), ↓Tumor necrosis factor-alpha (TNF-α)) and amplifies endogenous antioxidant defenses (↑Superoxide dismutase (SOD), ↑Glutathione (GSH)) [15, 35].

Clinically, these integrated pathways result in significant cardiovascular enhancements. Engaging in moderate-to-vigorous intensity physical activity can lead to a reduction in systolic blood pressure by 5 to 8 mmHg and a decrease in Low-Density Lipoprotein (LDL) cholesterol by 10 to 15% among sedentary individuals. This is achieved through the optimization of lipid metabolism and the enhancement of the nitric oxide pathway [15, 37]. Intermittent high-intensity protocols significantly improve the efficiency of myocardial oxygen utilization and reduce arterial stiffness. These combined effects help lower the risks associated with hypertension and atherosclerosis [15, 40].

### 2.5 From Research to Clinical Practice and Public Health Promotion

IPA provides an effective approach to overcoming the obstacles linked to structured exercise, such as limited time and the requirement for specialized equipment. It presents accessible and practical movement options tailored for sedentary groups, including office workers, older adults, and those with CVDs [41, 42].

Core strategies for implementation include integrating activities such as stair climbing, active breaks, and household chores into daily routines [9, 14]. Clinicians can encourage patients to adopt several practical strategies to increase their physical activity. These include parking further away from their destinations, opting for stairs rather than elevators for trips of three floors or less, and scheduling “walking meetings” or taking active breaks every 30 to 60 minutes during prolonged periods of sitting. Additionally, engaging in household chores like vacuuming or gardening at a brisk pace. The WHO’s recommendation of 150 minutes of moderate-to-vigorous physical activity (MVPA) per week can be achieved through accumulated incidental physical activity (IPA). For instance, this may include 10 minutes of brisk walking for daily transport (moderate-intensity IPA), 15 minutes of vigorous gardening (vigorous-intensity IPA), and 5 minutes of stair climbing (vigorous-intensity IPA) distributed throughout the week. This lifestyle-integrated approach makes physical activity goals more attainable in everyday life.

While a minimum of 45 minutes per week of VIPA correlates with optimal mortality benefits, it is important to exercise caution when recommending VIPA to patients with unstable CVDs due to its potential arrhythmogenic effects [8, 43]. Therefore, promoting IPA requires tiered recommendations based on the principle of “graded health benefits”. The practical application of IPA should focus on personalized prescriptions and risk stratification. For example, frail individuals or those with advanced CVDs should receive customized adjustments to their programs to prevent overexertion [44]. For high-risk groups (e.g., older adults, individuals with metabolic syndrome, stable CVD), pre-participation cardiovascular screening (e.g., history, physical exam, Electrocardiogram (ECG), or stress testing for silent ischemia) is recommended before engaging in VIPA [8, 15, 23, 45]. Based on sports cardiology guidelines [23], initial intensity should be moderated (e.g., staying below 85% of age-predicted maximum heart rate, Borg Scale perceived exertion <15/20) and progressed gradually. Supervised cardiac rehabilitation programs are the gold standard and essential starting points for these populations [8], where the benefit-risk ratio of unsupervised VIPA can be carefully evaluated.

The safe implementation of IPA requires a careful and gradual transition from low-intensity to high-intensity exercise sessions. It is crucial to monitor perceived exertion in real-time, using tools such as the Borg Scale and hemodynamic parameters. This approach helps to mitigate the risks of developing arrhythmias or experiencing exertional angina during physical activity [8, 15, 46]. The quantification of sporadic, low-volume IPA bouts presents a challenge, as self-report questionnaires (e.g., IPEQ) are prone to overestimating activity intensity [25, 47], while wrist-worn accelerometers may underestimate certain activities, such as cycling or weight-bearing tasks [9, 24, 48]. Therefore, multimodal assessments integrating accelerometry, heart rate monitoring, and machine learning classifiers are essential to enhance the accuracy of IPA measurement [49]. Clinicians should prioritize wearable technologies for personalized activity prescriptions [50].

To achieve sustained long-term engagement, time-efficient approaches such as “exercise snacks” (e.g., 5-minute stair climbing) are recommended [15, 19]. Furthermore, AI-driven applications for real-time feedback may also be used to enhance adherence [8]. Environmental interventions, such as stair-use prompts, can increase participation by 12–15% [51], and urban planning initiatives that emphasize mixed-use zoning and pedestrian connectivity may potentially enhance active transportation [52]. For sedentary populations, vigorous intermittent lifestyle physical activity and workplace interventions are effective strategies to integrate movement into daily routines [53].

### 2.6 Limitation

Current evidence highlighting the cardiovascular benefits of IPA largely stems from studies conducted on non-exercising adults within the UK Biobank. However, this evidence base has limitations due to the cohort’s lack of representativeness, which may skew effect estimates. Notably, over 90% of participants identify as White, contrasting sharply with the more diverse general population. Additionally, the educational attainment within this cohort is significantly higher, with over 55% holding university degrees compared to approximately 27% nationally. Socioeconomic factors also play a role, as participants tend to live in less deprived areas and engage in healthier behaviours, likely leading to an attenuation of risk estimates and an overestimation of the protective effects of physical activity. Moreover, the cohort is predominantly older, with ages ranging from 40 to 79 at the time of recruitment, resulting in a lack of representation for younger and very elderly groups. The “healthy volunteer” effect further exacerbates this issue by excluding individuals with high genetic susceptibility, severe comorbidities, or adverse socioeconomic conditions. Consequently, these biases may result in an underestimation of risk associations and limit the generalizability of findings to more diverse, disadvantaged, or high-risk populations [24, 54]. Consequently, future research should prioritize longitudinal studies encompassing diverse demographic groups and further focus on refining tools for accurately measuring IPA [12].

## 3. Conclusion

This review enhances the field of cardiovascular prevention science by proposing a structured classification system for incidental physical activity (IPA) based on exercise intensity, while also synthesizing current evidence concerning its health effects. Our methodology introduces an operational definition that prioritizes behavioural context over motivation, effectively addressing previous inconsistencies in the concept. A significant novel contribution of this work is the quantification of intensity equivalence; we demonstrate that one minute of vigorous IPA is roughly equivalent to three minutes of moderate IPA in terms of cardiovascular risk reduction. From a public health perspective, these findings underscore the potential of IPA as an accessible solution to overcome common physical activity barriers, particularly among sedentary groups, older adults, office workers, and those with cardiovascular conditions. Incorporating IPA into daily routines offers a scalable, low-resource strategy for reducing population-level cardiovascular risk. The available evidence reinforces the position of IPA as a feasible and effective public health intervention, supporting its inclusion in clinical guidelines and wider health initiatives.

## Abbreviations

CI, confidence interval; CVD, cardiovascular disease; HR, hazard ratio; IPA, incidental physical activity; LIPA, light intensity IPA; MACE, major adverse cardiovascular events; MET, metabolic equivalent of task; MIPA, moderate intensity IPA; MV-ILPA, moderate-to-vigorous intermittent lifestyle physical activity; PAF, population-attributable fraction; VIPA, vigorous intensity IPA; VO_2_, oxygen uptake.
